# Extending the detection limit: innovations in infrared quantum dot photodetectors reaching up to 18 μm

**DOI:** 10.1038/s41377-024-01504-3

**Published:** 2024-07-08

**Authors:** Chong Wu Wang, Qi Jie Wang

**Affiliations:** 1 Centre for OptoElectronics and Biophotonics, School of Electrical and Electronic Engineering, Nanyang Technological University, Singapore, 639798 Singapore; 2 Centre for Disruptive Photonic Technologies, School of Physical and Mathematical Sciences, Nanyang Technological University, Singapore, Singapore

**Keywords:** Quantum dots, Optoelectronic devices and components

## Abstract

A regrowth method was used to synthesize large-sized colloidal quantum dots (CQDs). With the assistance of doping engineering, the synthesized CQD detectors demonstrate exceptional long-wavelength infrared detection performance, reaching up to 18 μm, significantly extending the spectral response limit for CQD-based infrared detectors. These detectors also achieve a reasonably high detectivity of 6.6 × 10^8^ Jones.

Photodetectors, which convert optical signals into electrical signals, are fundamental and significant devices for various applications, including imaging, spectroscopy, security, optical communication, etc. Traditionally, photodetectors are made from bulk semiconductors. For example, materials such as GaN, Si, InGaAs, and HgCdTe (MCT) have been investigated for detecting light from the ultraviolet to the mid-infrared range^1–4^. Since the first synthesis of quantum dots in 1983^5^, these nanomaterials have shown great potential as promising candidates for photodetectors due to their remarkable flexibility, high absorption, low cost, and ease of fabrication^6–9^. Notably, the 2023 Chemistry Nobel Prize was awarded to Alexei Ekimov, Louis Brus, and Moungi Bawendi for their pioneering work on the discovery and synthesis of quantum dots^10^.

The spectral response of the quantum dot detectors can be actively tuned by engineering their bandgaps and modifying their sizes and compositions^11^. For instance, Ding, etc. demonstrated a ligand exchange method to fabricate hybrid Er^3+^ doped perovskite quantum dots, achieving broadband photodetection from the ultraviolet to the near-infrared^12^. Zhang et al. synthesized n-type HgTe QDs for intraband transition in the long-wavelength infrared (LWIR) region, reaching up to 12 μm, with a response of approximately 1 × 10^-3^ A/W and a detectivity of ~1 × 10^7^ Jones at 80 K^13^. However, unlike bulk MCT infrared detectors, which can respond up to 25 μm, it remains challenging for QD-based infrared detectors to have photoresponse in the very long-wavelength infrared (VLWIR) region (above 12 μm).

In a newly published paper in *Light: Science & Applications*, Chen Menglu and co-authors from the School of Optics and Photonics, Beijing Institute of Technology, have proposed and demonstrated a regrowth method combined with ionic doping modification to synthesize large-size HgTe quantum dot for efficient VLWIR detection up to 18 μm^14^. Large-size HgTe CQD exhibits responses of 0.3 A/W and 0.13 A/W, with detectivities of 6.6 × 10^8^ Jones and 2.3 × 10^9^ Jones for mid-infrared detections at 18 μm and 10 μm, respectively, at 80 K. These results not only extend the wavelength limit for CQD-based detectors but also inspire further interest in bottom-up fabricated infrared detectors, offering a cost-effective alternative to traditional epitaxially-grown semiconductor bulk detectors.

Fabricating large-sized quantum dots is challenging due to their poor colloidal stability. Typically, synthesizing large-sized HgTe colloidal quantum dots requires an excess amount of mercury precursor, with mercury ions (Hg^2+^) acting as surface ligands to ensure good stability. In this study, the ratio of mercury to tellurium is 4:1. Additionally, the researchers developed a regrowth method (Fig. 1a) using highly active trimethylsilyl telluride (TMSTe) for rapid nucleation, followed by the gradual addition of low-active trioctylphosphine telluride (TOPTe) for nanocrystal regrowth.

Another obstacle for VLWIR detection using CQD is the short carrier drift length, typically only spanning several quantum dots due to the narrow bandgap. As a result, the performance of the CQD infrared detector drops rapidly in the LWIR region and usually leads to no response in the VLWIR region. To improve performance (Fig. 1b), the authors propose ligand exchange and precise doping control using an iodine treatment. (Fig. 1c) The treated CQDs show a carrier mobility of around 10 cm^2^/Vs, which is around 100 times higher than that of pristine CQDs. Consequently, the photoresponse increases drastically from 0.2 mA/W to 0.13 A/W at 10 μm detection, corresponding to a 670-fold enhancement in external quantum efficiency. Additionally, the authors found that the responsivity is limited by the ratio of carrier drift length to the electrode gap and the absorption coefficient. These findings indicate that appropriate ligand modifications to achieve high mobility and good carrier lifetime are crucial for LWIR and VLWIR detection (Fig. 1).

**Fig. 1:**
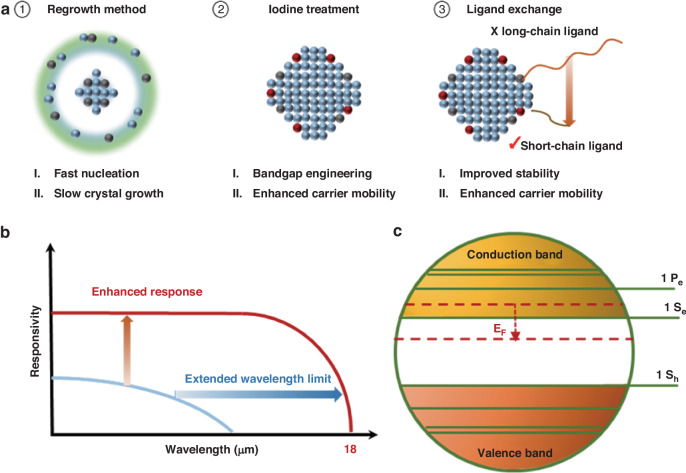
Schematic illustration for the VLWIR detection (up to 18 μm) with large size HgTe quantum dots. **a** Schematic illustrations for the synthesis strategies used for fabricating large-sized quantum dots for very long-wavelength infrared (VLWIR) detection. **b** The demonstrated detectors with the fabricated QDs show enhanced photoresponse and extended wavelength limit. **c** The energy diagram for I_2_-treated QDs

Beyond exploring the wavelength limitations of CQDs, it is equally important to improve other detection parameters such as detectivity and operational temperature. Hao Qun and colleagues from the Beijing Institute of Technology have demonstrated the first high operation temperature (HOT) CQD device using a mixed-phase ligand exchange method^15^. In traditional bulk semiconductor detectors, Prof Carlo Sirtori introduced and demonstrated room-temperature operation for metasurface-coupled quantum well-infrared detectors with an antenna effect^16^. Adopting a similar strategy in CQD infrared detectors could be promising. Additionally, upconversion nanoparticles, which convert mid-infrared light into visible or near-infrared light, offer great potential for room-temperature infrared detection using CMOS-compatible Si-based photodetectors. Recently, the groups led by Liu Xiaogang and Wang Qi Jie jointly demonstrated the Nd^3+^ doped NaYF_4_ nanocrystals for efficient Mid-Infrared (MIR) upconversion detections and applied them to MIR spectroscopy and imaging^17^. This finding also paves a new pathway for high-performance MIR detection.

Overall, the future of quantum dot detectors is bright, with exciting potential for continued innovation, expanded applications, and commercialization. By overcoming technical hurdles and harnessing the full potential of quantum dots, these detectors may revolutionize sensing capabilities across a wide range of fields.
